# Sunsetting Binding MOAD with its last data update and the addition of 3D-ligand polypharmacology tools

**DOI:** 10.1038/s41598-023-29996-w

**Published:** 2023-02-21

**Authors:** Swapnil Wagle, Richard D. Smith, Anthony J. Dominic, Debarati DasGupta, Sunil Kumar Tripathi, Heather A. Carlson

**Affiliations:** Department of Medicinal Chemistry, College of Pharmacy, University of Michigan, 428 Church St, Ann Arbor, MI 48109-1065 USA

**Keywords:** Biophysics, Computational biology and bioinformatics, Structural biology

## Abstract

Binding MOAD is a database of protein–ligand complexes and their affinities with many structured relationships across the dataset. The project has been in development for over 20 years, but now, the time has come to bring it to a close. Currently, the database contains 41,409 structures with affinity coverage for 15,223 (37%) complexes. The website BindingMOAD.org provides numerous tools for polypharmacology exploration. Current relationships include links for structures with sequence similarity, 2D ligand similarity, and binding-site similarity. In this last update, we have added 3D ligand similarity using ROCS to identify ligands which may not necessarily be similar in two dimensions but can occupy the same three-dimensional space. For the 20,387 different ligands present in the database, a total of 1,320,511 3D-shape matches between the ligands were added. Examples of the utility of 3D-shape matching in polypharmacology are presented. Finally, plans for future access to the project data are outlined.

## Introduction

Databases of protein–ligand complexes are central to various drug discovery and design projects. They are particularly useful in polypharmacology projects, such as predicting off-target activities of drugs (toxicology) or finding novel applications of known drugs (drug repurposing). There are several databases that provide data on protein–ligand complexes, including Binding MOAD (www.BindingMOAD.org)^1–3^, PDBbind (www.PDBbind.org.cn)^4^, BindingDB (www.bindingdb.org)^5–9^, sc-PDB (http://bioinfo-pharma.u-strasbg.fr/scPDB/)^10,11^, and many more. These databases are aimed at different applications, and their content and sizes vary because of their different selection criteria for including any particular protein–ligand complex.

MOAD was initiated in 2001, first published in 2005^1^, and annually updated in early January of each year. When we began MOAD, the largest datasets for docking and scoring had roughly 200 complexes^12,13^. These were gathered in a “bottom up” approach of reading the medicinal chemistry literature to identify structures. We decided to use a “top down” approach that started with the whole Protein Data Bank (PDB) containing all possible complexes and augment that maximal set with affinity data through literature searching. A protein–ligand complex must have a resolution of at least 2.5 Å and contain at least one biologically relevant ligand in its PDB structure to be included in the database. In our HiQ subset of pristine protein–ligand complexes from MOAD^14^, additional selection criteria require more exacting metrics of R_free_ − R_work_ ≤ 5%, Real Space R ≤ 0.2, and RSCC ≥ 0.9.

In 2014, the website and database were restructured into a LAMP (Linux, Apache, MySQL, and PHP) format^3^; the improved user interface incorporated third-party plugins, such as Jmol, MarvinView, and JChemBase with MarvinSketch for better visualization of proteins and ligands. In the same update, useful features like filtered downloads and field-based searching were also incorporated. In 2019, NGL viewer was added for an improved visualization of the protein–ligand complexes, and MarvinView was replaced with MarvinJS for small-molecule searching in the database^15^. The website was also equipped with polypharmacology tools, such as 3D binding-site similarity and 2D similarities of ligands.

Our latest addition to MOAD is 3D similarities across the ligands. Similar molecules tend to have similar chemical and biological properties^16^. Assessment of structural similarities among small molecules can be a highly effective starting point for the discovery and optimization of various lead molecules. This is useful in predicting toxicological properties of off-target binding and repurposing drugs as potential inhibitors to other proteins of interest. Two-dimensional molecular similarity approaches have been quite popular because of their simplicity and accuracy^17–22^. However, 2D similarity calculations are mostly based on molecular fingerprint descriptors and do not contain any information about the 3D structure of a molecule. We chose to add molecular 3D similarity because of its importance in virtual screening of molecular libraries as well as scaffold hopping approaches^23–25^.

Our consistent efforts in updating MOAD has made the database a popular choice among scientists. MOAD’s papers have been cited 600 times, and the website receives ~ 1000 hits a week. Many recent machine learning studies have introduced novel scoring functions for molecular docking^26,27^ based on MOAD as a benchmark set. In a recent study, MOAD served as a benchmark set for RosENET (Ro**s**etta Energy Neural Networks), a three-dimensional convolutional neural networks based study that combined molecular mechanics energies and descriptors for predicting the absolute binding affinity of protein–ligand complexes^28^. The high quality of protein–ligand structures in the database also made MOAD a popular choice for some other neural network based studies, such as K_DEEP_^29^, DEELIG^30^, and DeepAtom^31^. These studies used three-dimensional voxelized representation of protein–ligand complexes for extractions of molecular features and binding related interaction patterns, in order to predict the binding affinities of the complexes. High quality of the structures of the complexes seems to be crucial for the success of the voxelized representation of complex structures. Historically, MOAD has been used to develop and test molecular mechanics parameters and docking and scoring methods^32–47^; to examine fundamental protein–ligand interactions^48–58^; to predict small molecules ligands, protein targets, and binding sites^59–62^; and to aid protein design^63,64^. Our own efforts with MOAD have focused on learning biophysical principles behind protein–ligand binding and relating those patterns to affinity^65–68^. Furthermore, we used MOAD’s data to hold the first docking and scoring contests in the field^14,69^.

## Methods

As noted above, the data collection in MOAD is performed using a “top-down” approach, i.e., first all the protein–ligand complex structures from the RCSB Protein Data Bank (https://www.rcsb.org/) are imported, then the structures that do not satisfy the inclusion criteria of MOAD are discarded, and finally the binding data for the included PDBs are extracted from the primary crystallography references. The primary reference is the reported reference for the PDB structure in the RCSB Protein Data Bank (PDB)^70^.

The RCSB databank had 160,152 protein–ligand complex structures on 1/2/2021, which were imported to our data pipeline for inspection. A total of 1078 journal articles were acquired for assessing the new structures added in the previous year. A detailed description of the procedure for the data pipeline has been reported in previous MOAD updates^1–3,15^. An abbreviated summary of the pipeline is as follows:

1. Structures with resolution worse than 2.5 Å are discarded. The remaining structures are checked for at least one protein chain and at least one ligand that is not bound to the protein chain covalently.
2. The ligand(s) in each of the structures is checked for its biological relevance and annotated as “valid,” “invalid,” or “part of the protein.” Crystallographic additives, salts, buffers, metals, and solvents are considered invalid. HEME groups and modified amino acids in the protein chains are considered part of protein and not bound ligands.
3. Structures emerging from the step 2 with at least one valid ligand are hand curated before their final entry into MOAD. No structure is included in the database without being manually inspected. The binding data, whenever available, is extracted from the primary references for the crystal structure. Whenever multiple kinds of binding information are reported, our order of preference for selecting the data is *K*_d_ > *K*_i_ > IC_50_ (dissociation (or association K_a_) constants over inhibition constants over half-maximal inhibitory concentrations).

The protein–ligand complexes are then grouped into families based on sequence similarities, which are calculated using BLAST^71^. A family contains all the complexes in the database that have sequences ≥ 90% identical to each other. Each of the families is assigned a leader that is typically the complex with the tightest binding ligand in the set. When binding data is not present for any member of the family, the leader is selected based on the ligand that has the best resolution and most biological relevance. Proteins are also grouped on 50% and 70% sequence similarities as well, if researchers prefer to analyze homologous sets of proteins, although no leader is chosen for these groups.

### Cross comparison of affinity data

A unique feature of this last update is that the binding data in MOAD was cross-referenced with that of PDBbind^4^. Each of the collected discrepancies were checked manually for the correct value of the binding data from the primary reference of the PDB entry. If MOAD values were incorrect, the correct values were added to the database, and those are now available on the database website. A detailed analysis appears below in Results and Discussion.

### 3D ligand similarity

Calculations of 3D-shape similarity were performed using ROCS^23^ and FastROCS^23,24^ from OpenEye, based on all the valid ligands of MOAD database. Although ROCS has the utility to also perform color (or chemical feature) similarity search, it was not used in our analysis for tractability.

ROCS calculations are based on the concept that two entities will have the same shape if their volumes exactly correspond. Therefore, for any two overlaid ligands, the volume mismatch is a measure of dissimilarity. The converse of this is not true, i.e., two objects that have the same volume do not necessarily have the same shape. In the shape theory of ROCS, the precise definition of shape similarity between two objects is given by the integral

$$S_{1} = \int {\left| {f\left( {x, y, z} \right) - g\left( {x, y, z} \right)} \right|dV}$$

where $f\left( {x,y,z} \right)$ and $g\left( {x,y,z} \right)$ are the characteristic functions of the objects. Molecular volume is represented by smooth Gaussians rather than hard spheres. ROCS uses a solid-body optimization process for molecules that maximizes the overlap between two molecules.

As an abstract definition, a Tanimoto coefficient is the ratio of the intersection and the union of two sets. In ROCS, the Tanimoto coefficient of the object is calculated by the equation

$$Tanimoto_{f,g} = \frac{{O_{f,g} }}{{I_{f} + I_{g} - O_{f,g} }}$$

where the $I$ terms correspond to self-volume overlaps and $O$ term corresponds to the overlap between the two characteristic functions.

In our study, every ligand in MOAD was taken as a query ligand and compared to all the other ligands of the database. Comparisons were ranked based on their volume alignment between the query ligand and the alternate ligand. For a given ligand pair, first starting from the centers of mass of the ligands, the ligands were superimposed and a Tanimoto coefficient was calculated. A second Tanimoto coefficient was also calculated by taking the maximum Tanimoto coefficient over 8 different overlapping superpositions. The additional overlapping structures were generated by placing the center of mass of the query molecule randomly over the second molecule and optimizing for maximum volume overlap. This was done to account for the variable sizes of the ligands. All the conformations were kept rigid during all similarity calculations.

FastROCS is a tool from OpenEye that performs 3D similarity calculations using GPUs. The shape theory behind FastROCS is the same as that of ROCS, though FastROCS uses a slightly different algorithm to calculate molecular overlaps due to a modified GPU-version of the computer code. The maximum of three Tanimoto coefficients (ROCS in which center of masses of the query and database molecules were aligned, ROCS in which 8 conformations were generated by placing the center of mass of the query molecule randomly over the database molecule were generated, and FastROCS) was taken as the final Tanimoto coefficient for the query-database pair.

The abovementioned calculations were performed over the PDB conformations of the ligands. However, it is possible that a ligand can be very similar to a query ligand in a conformation that is not reported in its PDB structure but is nevertheless energetically favorable. Therefore, it is important to find ligands which might exhibit high shape similarity with a PDB conformation of one ligand, when the Tanimoto coefficient is calculated with a different conformation of the second ligand. Therefore, 64 different conformations of all the unique valid ligands in MOAD were generated using OpenEye Omega^72,73^. Prior to conformation generation, the ligands were passed through the Filter utility of OpenEye to eliminate undesirable compounds to save execution time. In the Filter screening of the valid ligands, checks on Lipinski violations were removed, limits on constraining the physical properties were relaxed, and the Boolean flag for constraining pH = 7.4 (-pkanorm) was set to false. The ligands were then separated into macrocycle and non-macrocycle molecules, as Omega uses different methods to generate conformations for the two. For non-macrocycle molecules, conformations were generated using Omega’s ‘fastrocs’ mode. For each of the macrocycle and non-macrocycle molecules, a maximum of 64 different conformations were generated, whenever possible.

Shape similarity calculations were performed between all the generated conformations (as database ligands) and the PDB-reported conformations of all the ligands (as query ligands). The total number of unique conformations generated for these calculations was ~ 950,000. In this study, we performed a total of 16 billion ROCS and 85 billion FastROCS calculations.

## Results and discussion

### The update

The current update of MOAD contains a total of 41,409 valid protein–ligand complex structures, which is a 26% increase since the last MOAD communication in 2019^15^. The valid protein–ligand complexes are grouped into 11,058 protein families and collectively contain a total of 20,387 unique ligands. A total of 15,223 binding data entries are reported for the 41,409 complexes (37% coverage, which has been consistent over the last several years). The binding data entries contain 5,509 K_d_ values (including converted K_a_ data), 4,581 K_i_ values, and 5,131 IC_50_. Table 1 presents the ranges for each of the types of the reported binding data. Many ligands in MOAD have drug-like characteristics: 51% have affinities of 440 nM or better, 69.9% range 120–500 MW, and 69.4% have 0 or 1 Lipinski violation.

**Table 1:** The distribution of K_d_, K_i_, and IC_50_ in Binding MOAD.

	Tightest	Number of entries				Weakest
		< 1 nM	1 nM– 1 μM	1 μM–1 mM	> 1 mM	
K_d_ (or K_a_^−1^)	10.0 fM	323	2048	2796	342	K_a_ = 0.00108 M^−1^
K_i_	10.0 fM	540	2353	1468	220	837.0 mM
IC_50_	0.00316 nM	341	3235	1445	110	378.0 mM

### Comparing affinity values to PDBbind

PDBBind’s ‘refined set’ is a dataset comparable to MOAD^4^. The collection of the binding data for PDBbind is done through a keyword-based search through the full text of the primary reference provided in each relevant PDB structure, followed by independent manual examination of the text by two scientists who must agree on a value. With MOAD, the data extraction is done by basic natural language processing (NLP) and one person manually checking the primary reference of the PDB structure^2,74^. Our semi-automated, text-mining tool is BUDA (Binding Unstructured Data Analysis) that allows for guided reading to identify key sentences and phrases in papers; it has a weighted-scoring algorithm to rank the likelihood that sentences and phrases contain binding data. BUDA is a shared utility that allows coworkers to divide the structures among themselves and keep abreast of each other’s progress. The curators can sort the articles by their weighted scores, review texts with highlights noting key phrases or sentences, and update the data into Binding MOAD. The NLP portion of BUDA is built upon the General Architecture for Text Engineering (GATE) framework (gate.ac.uk). Our GATE pipeline consists of ANNIE plug-ins, modified lookup lists for its Gazetteer, multiple JAPE grammars, and processing/exporting tools. Our additions to the lookup lists include keywords like “dissociation constant”, “binding”, “IC_50_”, etc. The transducers annotate large phrases and sentences, eg. a transducer is used to group numbers and molar units (nM, mM, pM, etc.). A second transducer identifies and highlights patterns where a constant name is very near a number-unit pair. The BUDA dashboard displays each paper with highlights on the text, tables, and figure captions that help the curator find the needed information.

Since there are many structures common to both the MOAD and PDBbind databases, we performed a comparison of the binding data from the two sets and collected discrepancies. The discrepancies were reexamined again through manual inspection of the primary references of the PDB structures. Though PDBbind has adopted many of the quality protocols introduced in Binding MOAD over the years, this is the first time we have compared back to PDBbind.

The comparison between MOAD and the refined set of PDBbind resulted in a total of 2,371 disagreements. Most of the mismatches originated simply from a difference in preferences for reporting data in the literature. For example, MOAD emphasizes reporting dissociation (K_d_) and association (K_a_) constants over inhibition constants (K_i_) or half-maximal inhibitory concentrations (IC_50_). MOAD also aims to report data exactly as found in the literature, i.e., without changing the units of the data. Specifically, binding affinities for two-thirds of the complexes (1,589) were actually agreements with different units used (e.g., MOAD may report a literature value of 0.003 μM but PDBbind reports 3.0 nM). Of the 782 erroneous mismatches, MOAD contained 602 errors (making its error rate only 4% of the affinity entries collected over 20 + years). PDBbind contained the remaining 180 disagreements with the data in the crystallography papers, but it should be noted that differences with the values in PDBbind might stem from their search for affinity data outside the crystallography literature. The MOAD errors can be assigned to the following categories: 1) 98 with incorrect binding measurement type (e.g., MOAD reported K_d_ when the value was actually listed as either a K_a_, K_i_, or IC_50_); 2) 44 with incorrect inequality type (e.g., MOAD reported an “ = ” even though a “ ~ ” or a “ < ” was given in the literature); 3) 256 with an incorrect ligand interaction reported (e.g., data was reported for the wrong protein–ligand pair); 4) 204 cases of human error due to simply wrong reporting of the binding data. We are grateful for the excellent work in PDBbind curation that made this error check possible.

### Adding 3D similarity

**Figure 1:**
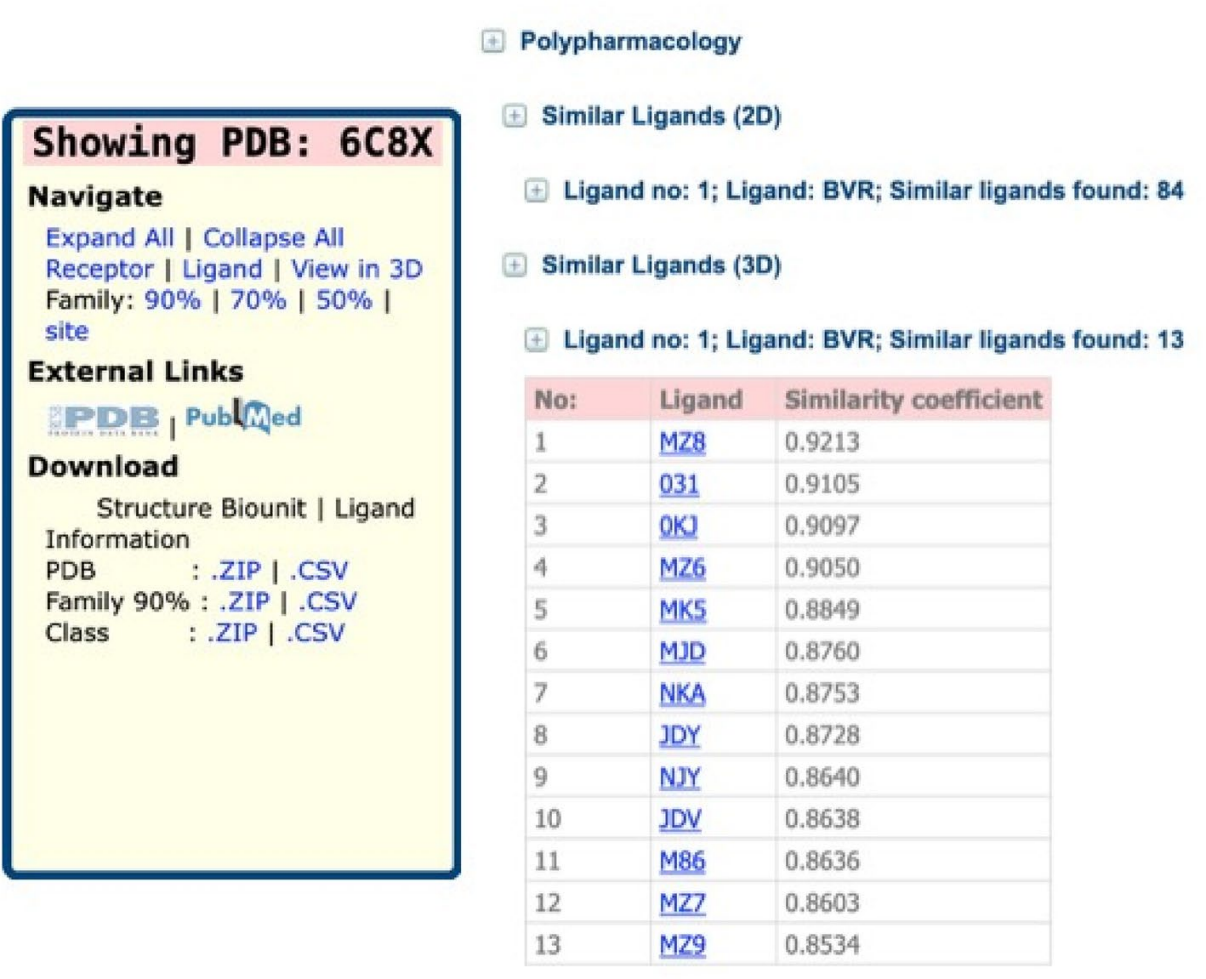
Example of the 3D similarity of an entry on BindingMOAD.org.

Our most recent features in MOAD have added polypharmacology tools. Polypharmacology is the binding of one small molecule at multiple target proteins. Off-target activities of small molecules (toxicology) and finding novel applications of known drugs (drug repurposing) are typical applications of polypharmacology. The relationship between shapes of protein binding sites and shapes of the ligands that bind to them is not one to one or one to many but is many to many. A recent study by Gao and Skolnick showed that the protein binding sites and ligands interaction are rather complicated because of promiscuous natures of protein binding sites as well as that of ligands^75^. The study also points out that the shape space of protein binding sites is finite and can be represented by about 1000 pocket shapes^76,77^. A significant set of shape features in a binding site can therefore be found in another binding site, which may not share any evolutionary relationship. As such, it is important to investigate polypharmacology prospects of a ligand by comparing its shape not only with other ligands in the same protein family but throughout the database. Ligand promiscuity also indicates that different conformations of a ligand might result in its binding to different binding sites^78^. Therefore, we have investigated 3D similarities of the ligand conformations with the known poses of a protein bound ligand (reported in PDB structures). The 3D similarity calculations conducted for the ligands of MOAD have enhanced the identification of small molecules with potential polypharmacological properties. The 3D similarity pairs are reported on the website for Tanimoto similarity > 0.85. From the similarity calculations performed on the ~ 950,000 ligand conformations (PDB-reported and those generated by Omega), more than 26 million individual similarities across all the conformations were identified and a total of 1,320,511 new 3D-shape matches between the individual ligands were added to the MOAD database. These have been added to a new 3D similarity section for ligands on our BindingMOAD.org pages for each complex, see Fig. 1.

### Examples of using 3D-ligand similarity from the Binding MOAD resource

**Figure 2:**
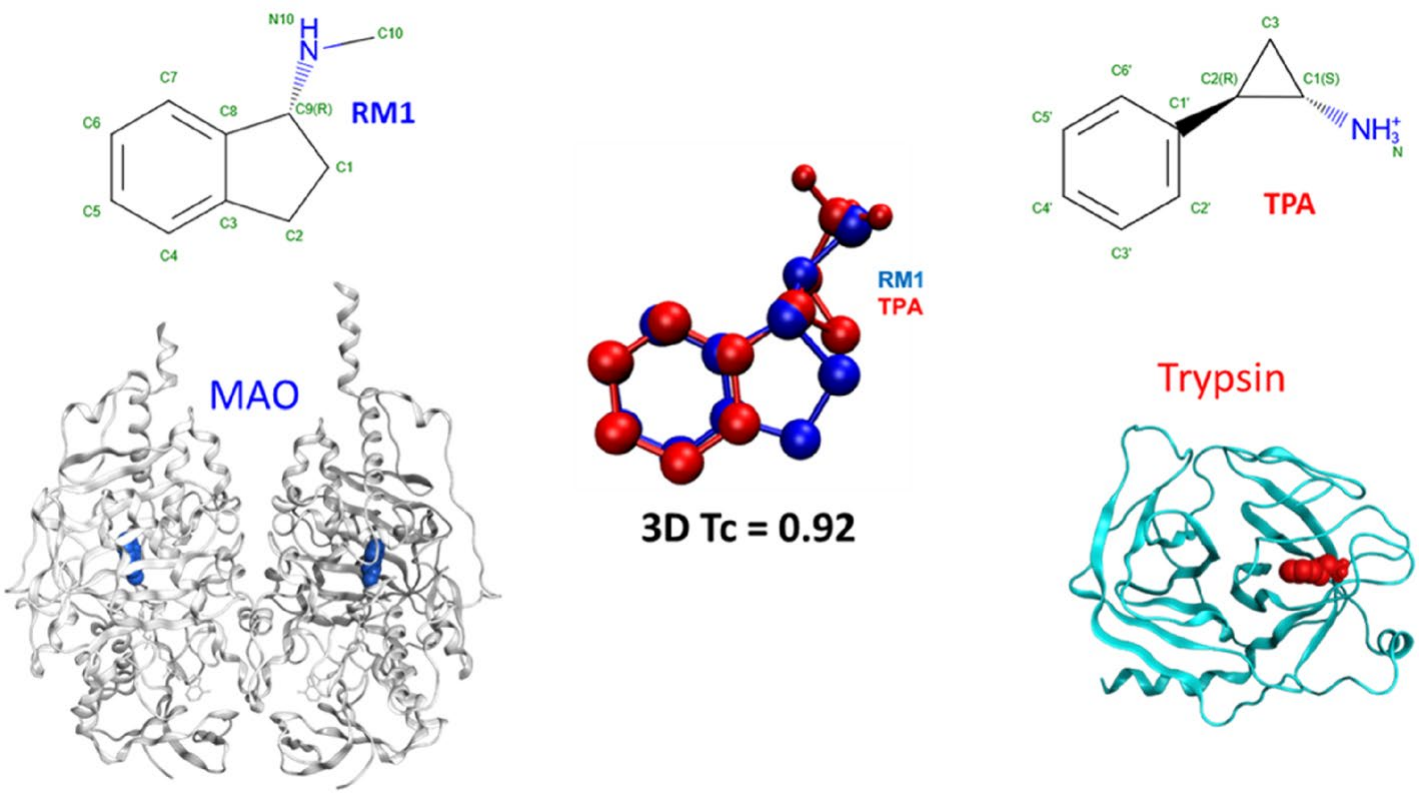
TPA has little 2D similarity to RM1, and its binding site in trypsin has no similarity to the binding site of MAO. However, their 3D similarity shows the connection of TPA as an inhibitor of MOA.

The new 3D matches in MOAD can identify off-target activities of small molecules as well as potential applications of known drugs. Figure 2 shows such an example; the molecule N-Methyl-1(R)-Aminoindan (RM1) is a rasagiline analogue that shows inhibitory activity to the protein monoamine oxidase (MAO, PDBid: 2C67)^79^ whereas the molecule Tranylcypromine (TPA) is an inhibitor for the serine protease trypsin (PDBid: 1TNL)^80^. The two molecules are not similar by a 2D comparison, and their binding sites do not match according to APOC or GLoSA. However, RM1 and TPA have a 3D Tanimoto coefficient of 0.92, revealing a very similar shape. We searched the literature and indeed found that TPA can be effective against MAO, a target for clinical depression^81^.

**Figure 3:**
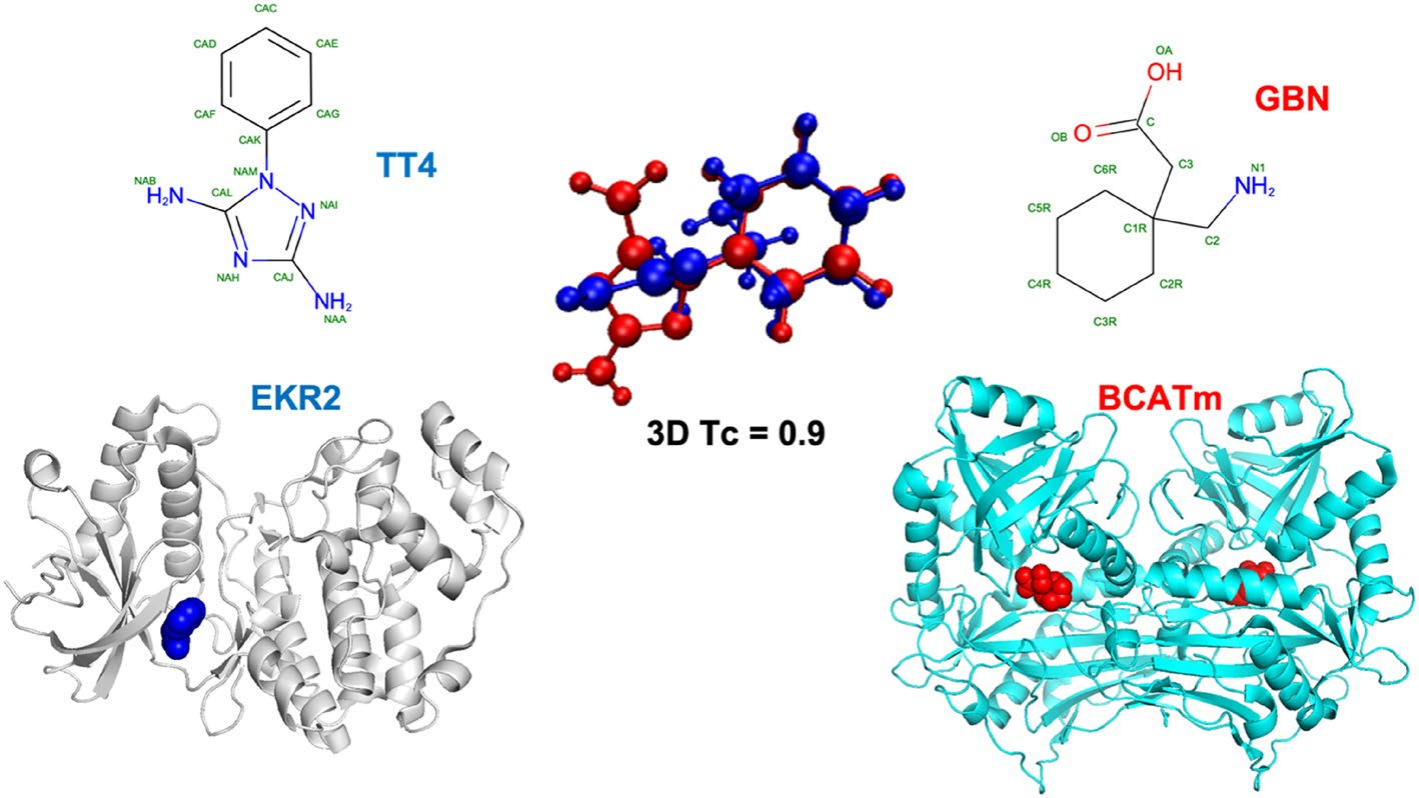
The 3D similarity of TT4 and GBN is evident. However, 2D similarity is low and their binding sites show no similarity as well.

Another example can be seen in Fig. 3. The molecule 1-phenyl-1H-1,2,4-triazole-3,5-diamine (TT4) binds to mitogen-activated protein kinase 1 (also known as ERK2, PDBid: 4XNE) in rats (*Rattus Norvegicus*)^82^. Neuroactive drug Gabapentin (GBN) is a molecule shown to form complex with human mitochondrial branched chain aminotransferase (BCATm, PDBid: 2A1H)^83^. Despite there being no similarity of the molecules by 2D measures and no similarity of the binding sites, it was found that GBN can inhibit ERK2 in rats^84^. The 3D Tanimoto coefficient for the two ligands in our calculations was 0.90.

## Conclusions

Here, we report the last update of the Binding MOAD database. While the addition of 3D-ligand similarity calculations is a powerful benefit, searching through all the ligand comparisons has significantly slowed the time for loading pages at the website, which is problematic.

The options available on the website include downloading the entire dataset, filtered downloads, ligand-based searches (though MarvinSketch), and an individual webpage for each of its complexes (identified by their PDBid). Each complex’s webpage is equipped with the family’s annotation (90%, 70% and 50% sequence similarities) with other proteins in the database. Similarity calculations (2D and 3D) for each of its ligands and binding-site similarity are also annotated.

The database will continue to be available online at BindingMOAD.org for another year and a half (through June 31st, 2024 when the server’s operating system will no longer be supported). For future access, the binding data will continue to be available via the RCSB PDB website pages for each complex. The backend of the website, including all affinity data and polypharmacology relationships across the dataset has been licensed to Chemical Abstract Services.

## Data Availability

The data in this manuscript are available at www.BindingMOAD.org and the RCSB Protein Data Bank (www.rcsb.org).
